# Composition and Antioxidant Properties of Pigments of Mediterranean Herbs and Spices as Affected by Different Extraction Methods

**DOI:** 10.3390/foods10102477

**Published:** 2021-10-16

**Authors:** Daniela Cvitković, Patricija Lisica, Zoran Zorić, Maja Repajić, Sandra Pedisić, Verica Dragović-Uzelac, Sandra Balbino

**Affiliations:** Faculty of Food Technology and Biotechnology, University of Zagreb Pierottijeva 6, 10000 Zagreb, Croatia; plisica@pbf.hr (P.L.); zzoric@pbf.hr (Z.Z.); maja.repajic@pbf.unizg.hr (M.R.); sandra.pedisic@pbf.unizg.hr (S.P.); vdragov@pbf.hr (V.D.-U.); snedjer@pbf.hr (S.B.)

**Keywords:** Mediterranean plants, pigments, antioxidant capacity, pressurized liquid extraction, agitation-assisted extraction, ultrasound-assisted extraction

## Abstract

This study examined the composition and properties of chlorophyll and carotenoid extracted from the leaves of several Mediterranean evergreen shrubs and subshrubs (*Myrtus communis* L., *Pistacia lentiscus* L., *Thymus vulgaris* L., *Salvia officinalis* L. and *Laurus nobilis* L.) commonly used as herbs and spices. In order to fully assess their composition over a wide polarity range, pigments were extracted by successive solvent extraction with hexane, 80% acetone and 96% ethanol. Agitation-assisted extraction (AAE), ultrasound-assisted extraction (UAE) and pressurized liquid extraction (PLE) were employed and compared regarding their effect on the pigments’ yield and composition. Individual chlorophylls and carotenoids were analyzed by HPLC-DAD, while the content of total pigments and the extracts’ antioxidant capacity were determined spectrophotometrically. Throughout the experiments, pheophytin *a*, *b* and *b*’ were dominant chlorophyll molecules, while lutein and β-carotene were dominant carotenoids. Overall, the extracted pigments were determined as being in the range of 73.84–127.60 mg 100 g^−1^ and were the lowest in *T. vulgaris,* with no significant differences between other species. *M. communis* and *P. lentiscus* had the highest antioxidant capacities, showing a moderate positive correlation with carotenoid and chlorophyll levels. Significant differences were found in the levels of individual pigments with most of them showing a medium level of polarity due to the dissolution in acetone as a medium polar solvent. AAE and PLE demonstrated similar efficacy in the extraction of both carotenoids and chlorophylls; however, preference can be given to PLE, being a novel method with numerous advantages, e.g., shorter extraction time and lower solvent consumption. The examined plant species certainly expressed great diversity and showed the potential for application in the production of various functional products.

## 1. Introduction

Mediterranean herbs are often used as condiments, i.e., sources of flavoring and coloring substances, as well as preservatives in food preparation. Besides their gastronomical usage, these herbs share a long tradition of application in folk medicine for the prevention and treatment of various gastrointestinal, urinary, dermatological, and inflammatory diseases, as well as injuries [1]. Evergreen shrubs and subshrubs are valued for their leaves, berries, and flowers, and hold an important place in the group of Mediterranean herbs. Typical representatives of these herbs are well-known members of various plant families: *Thymus vulgaris* and *Salvia officinalis* (Lamiaceae), *Laurus nobilis* (Lauraceae), *Myrtus communis* (Myrtaceae), and *Pistacia lentiscus* (Anacardiaceae) which are often utilized in the form of dried plant material, herbal infusions, syrups, or essential oils [2]. While *L. nobilis* and *T. vulgaris* leaves are commonly applied as spices and food preservatives [3,4], *M. communis* leaves are used in the perfume industry, and its berries are used for the preparation of liquors [5]. Mastic gum, obtained from *P. lentiscus* resin [6], and herbal tea from *S. officinalis* leaves [7] are also traditionally and widely used. Numerous studies have shown antioxidant, anti-inflammatory, antiproliferative, antimicrobial, insecticidal [8], antibacterial [3], antirheumatic, antiseptic [9], and anticarcinogenic [10] activity in these species and their extracts.

Lately, consumer preference for natural food additives has increased the global market demand for functional products obtained from natural sources [11], which implies a growing research interest in plants as one of the main sources of such additives. These include flavor enhancers [12] and coloring agents [13] that can successfully replace synthetic ones. In general, antioxidant capacity and the health benefits of herbs and their extracts can be attributed to numerous vitamins, polyphenols, and sterols, as well as pigments such as carotenoids, chlorophylls, and anthocyanins [14].

Chlorophylls are the most important lipid-soluble green plant pigments. Chlorophyll *a* (blue-green) and chlorophyll *b* (yellow-green) are characteristic of higher-order plants [15], with chlorophyll *a* playing a fundamental role and chlorophyll *b* an indirect role in photosynthesis [16]. Along with chlorophylls, carotenoids are also widely present pigments in the plant kingdom and are used as colorants and antioxidants by the food and pharmaceutical industries. The main subclasses of carotenoids are carotenes and xanthophylls. Lutein is the most abundant xanthophyll in plant leaves [17] while zeaxanthin is its isomer [18]. Lutein, zeaxanthin, and β-carotene are recognized as primary carotenoids responsible for photosynthesis [19]. Carotenoids and certain lipids protect chlorophylls in plants from light irradiation due to their photosensitivity [15].

Apart from the already mentioned antioxidative activity, these pigments possess other specific bioactive properties. Chlorophylls and their derivatives, including pheophytin, chlorophyllide and pheophorbide, also show strong anti-carcinogenic [20] and antimutagenic [21] activities. In addition, while chlorophylls are being scrutinized for their metal and toxin-binding bioactivity [22], the study by La Vecchia [23] showed that the increased intake of certain compounds (e.g., the carotenoids and flavonoids characteristic of the Mediterranean diet) is associated with the reduced risk of some types of cancer. Furthermore, carotenoids such as α- and β-carotene have shown provitamin-A activity [10]. Even though chlorophylls and carotenoids have been determined by several authors as appearing in various Mediterranean herbs [14,20,24], significant variations in results, and the lack of a systematic study that would include more species, limit the drawing of any conclusions about which herbs are, specifically, rich sources of these compounds. Besides, studies that employ HPLC determinations of individual pigments are even more limited and scarce [21,22,25]. Extraction is an essential step in the isolation of specific compounds from herbal matrices. Even though extraction efficiency depends on many internal and external factors, due to the well-known general principle of solubility that is based on the similar polarity of solvent and target molecules, the selection of extraction solvent is of primary importance. Consequently, groups of compounds that differ in their chemical structures are extracted using solvents of appropriate polarities. Successive (i.e., sequential) solvent extraction is based on the repetitive extraction and separation of the precipitate [23] from the supernatant, and the subsequent extraction (or re-extraction) of the precipitate with a solvent of different polarity. This type of extraction, therefore, offers a complete screening of the components of interest [26]. Öquist and Samuelsson [27] have shown a diversity of chlorophyll molecules obtained by the sequential extraction of *Pisum sativum* chloroplasts. The higher yield of chlorophyll *b* was obtained by the addition of a more polar solvent (ethanol) to a less polar solvent (petroleum ether), while chlorophyll *a* was primarily extracted in petroleum ether. Furthermore, the variation of antioxidant molecules, e.g., phenolic compounds, in different plant materials is often assessed by successive extractions [28,29].

Besides their selection, the economic use of solvents is also of great importance, due to their possible hazardous and toxic influence; thus, the potential of various advanced extraction techniques is being evaluated. Conventional methods, such as Soxhlet extraction and agitation-assisted extraction (AAE), are usually time-consuming, require larger amounts of solvents, and are limited by the number of samples that can simultaneously be manipulated. Therefore, advanced extraction techniques, such as microwave-assisted extraction, supercritical fluid extraction, ultrasound-assisted extraction (UAE), and pressurized liquid extraction (PLE), etc., are being utilized [30]. Even though these techniques have been extensively applied in the extraction of other compounds, mainly phenolics, their efficiency in the extraction of pigments has not been well established. Zlabur et al. [31] found that 20 min of UAE resulted in higher total chlorophylls and carotenoids of lemon balm and peppermint leaf extracts when compared to the conventional method. A study on carotenoid extraction from *Diospyros kaki* L., *Prunus persica* L., and *Prunus armeniaca* L. also showed that, when compared to the Soxhlet extraction, 5 min of extraction time in PLE proved to be a better choice [32].

Considering the mentioned lack of scientific data, this study aimed to examine and compare the chlorophyll and carotenoid profiles in the extracts obtained from leaves of selected Mediterranean shrubs and subshrubs (*M. communis*, *P. lentiscus*, *T. vulgaris*, *S. officinalis* and *L. nobilis*) that are widely used as spices and in food preparations. In order to fully assess the variety of pigment species present in these herbs, successive extraction using three solvents of different polarities (hexane, acetone (80%) and ethanol (96%)) was applied for the extraction. To assess and compare the efficiency of different extraction methods, AAE, UAE and PLE were also applied. HPLC-DAD analysis was used for the separation and detailed evaluation of chlorophyll and carotenoid content in the obtained extracts. Furthermore, chlorophyll *a*, chlorophyll *b*, and the total carotenoids were also analyzed spectrophotometrically, to determine the potential of the spectrophotometric method for the rapid screening of these pigments. The antioxidant capacity of the obtained extracts was monitored by the ferric reducing antioxidant power (FRAP) method.

## 2. Materials and Methods

### 2.1. Chemicals

Carotenoids (β-carotene, lutein, zeaxanthin), chlorophylls (chlorophyll *a*, chlorophyll *b*), iron-2,4,6-tripyridyl-s-triazine (TPTZ) and 6-hydroxy-2,5,7,8-tetramethylchroman-2-carboxylic acid (Trolox) were obtained from Sigma-Aldrich Chemie GmbH (Taufkirchen, Germany). All standards were at least 95% pure. Acetone, ethyl acetate, ethanol at 96%, and hexane were purchased from J. T. Baker (Phillipsburg, NJ, US). All chemicals and solvents were of HPLC grade.

### 2.2. Plant Material

Dry leaves of *P. lentiscus*, *T. vulgaris*, *S. officinalis*, *M. communis*, and *L. nobilis* were purchased from Suban Ltd. (Strmec Samoborski, Croatia). All samples were milled using an electric mill (WSG30, Waring Commercial, Torrington, CT, USA), sieved through a 2 mm sieve, and were immediately used for the extraction.

### 2.3. Extraction Conditions

Sample weight and solvent volume were adapted according to the demands of the individual method (AAE, PLE, UAE); however, their ratio was kept constant at 1:20 (2.5 g and 50 mL) for each experiment. All extractions were performed at 60 °C for 30 min in duplicates. Weighted samples were firstly extracted with hexane, and the obtained extracts were cooled down to room temperature, centrifuged at 5000× *g* rpm for 10 min (Hettich, Tuttlingen, Germany), filtered into a 50 mL volumetric flask, and made up to volume with the extraction solvent. Afterward, the residues were depleted twice more by successive extraction with solvents of increasing polarity (80% acetone and 96% ethanol, *v*/*v*), each time following the same extraction procedure described above. To enable the performance of the applied analytical methods, the obtained hexane supernatants were evaporated to dryness on a rotary evaporator (Heidolph Instruments GmBH & Co. KG, Schwabach, Germany) and dry extracts were dissolved in 50 mL of ethyl-acetate.

#### 2.3.1. Agitation-Assisted Extraction (AAE)

AAE experiments were performed using an agitation water bath (SBS40, Cole-Parmer, Stone, UK) according to the procedure described above at 120 rpm.

#### 2.3.2. Pressurized Liquid Extraction (PLE)

PLE was performed using a Dionex™ ASE™ 350 extraction system (Thermo Fisher Scientific Inc., Sunnyvale, CA, USA). The sample was mixed with diatomaceous earth (1.5 g) and added into a 34 mL stainless-steel extraction cell with two cellulose filters placed in the bottom (Dionex™ 350/150 Extraction Cell Filters, Thermo Fisher Scientific Inc., Sunnyvale, CA, USA). The extraction conditions were: 60 °C, 3 extraction cycles of 10 min static extraction time at constant pressure (10.34 MPa), 45% of flushing and 30 s of nitrogen purge. Further steps are as described in Section 2.3.

#### 2.3.3. Ultrasound-Assisted Extraction (UAE)

Falcon tubes (ISOLAB, Laborgeräte GmbH, Eschau, Germany) with sample and solvent were placed in an ultrasonic bath (Elma-Hans Schmidbauer GmbH & Co., Singen, Germany) set up with the following parameters: power level of 340 W, and frequency of 50–60 kHz at 60 °C for 30 min. Further steps are as described in Section 2.3.

### 2.4. HPLC-DAD Analysis

Carotenoid and chlorophyll contents were determined in all plant extracts (hexane, acetone, and ethanol) according to the method of Castro-Puyana et al. [33], with high-performance liquid chromatography (HPLC) using an Agilent 1260 Infinity quaternary LC system (Agilent Technologies, Santa Clara, CA, USA) equipped with a diode array detector (DAD). Develosil RP-Aqueous (C30) reversed-phase column (250 mm × 4.6 mm i.d., 5 µm particle size) (Phenomenex, Torrance, CA, USA) was used. A mixture of MeOH: methyl tert-butyl ether (MTBE): water (90:7:3, *v*/*v*/*v*) as mobile phase A and MeOH: MTBE (10:90, *v*/*v*) as mobile phase B was used. The flow rate was 0.8 mL/min^−1^ and the injection volume was 10 µL. The gradient of mobile phase elution was: 0 min, 0% B; 20 min, 30% B; 35 min, 50% B; 45 min, 80% B; 50 min, 100% B; 52 min, 0% B. Wavelength detection was at 450 and 660 nm. Standard solutions of β-carotene, lutein, zeaxanthin, chlorophyll *a*, and chlorophyll *b* were prepared for the calibration curve. The identification of chlorophyll derivatives was performed based on their DAD absorption spectra and relative retention times [34,35], while their quantification was based on the calibration curve of their original form (chlorophyll *a* or *b*). All results were expressed in mg per 100 g of dry weight (DW).

### 2.5. Spectrophotometric Determination of Chlorophylls and Carotenoids

The extracts were properly diluted, and their absorbance was determined using appropriate wavelengths, according to the solvent used. The following wavelengths were used for the determination of chlorophylls (*a*, *b*) concentration: 644 and 662 nm in ethyl acetate, 646.8 and 663.2 nm in 80% acetone, and 649 and 664 nm in ethanol extracts. In all extracts, 470 nm was used to determine the carotenoids.

Concentrations of these pigments were determined according to the Lichtenthaler and Buschmann [36] formulas and expressed in mg per 100 g of dry weight (DW).

### 2.6. The Ferric Reducing Antioxidant Power (FRAP)

According to Benzie and Strain method [37], the ferric reducing antioxidant power (FRAP) assay of obtained extracts was determined. This method is based on the reduction of iron-2,4,6-tripyridyl-s-triazine (TPTZ) into ferro-tripyridyltriazine. Briefly, 240 μL of distilled water, 80 μL of the sample, and 2080 μL of FRAP reagent was vortexed and thermostated for 5 min at 37 °C and the absorbance at 593 nm was measured. FRAP values are expressed as 6-hydroxy-2,5,7,8-tetramethylchroman-2-carboxylic acid (Trolox) equivalent (TE). The UV/Vis spectrophotometer (UV-1600PC, VWR, Radnor, PA, USA) was used for all spectrophotometric measurements.

### 2.7. Statistical Analysis

This research was based on a full factorial design, including the following independent factors: (i) plant species—5 levels, (ii) extraction method—3 levels, and (iii) solvent type—3 levels, with a total of 45 experimental conditions, each performed in two extractions (N = 90). All analytical determinations were carried out in two replications. The results were analyzed using a multifactorial analysis of variance (ANOVA), combined with Tukey’s multiple comparison tests (significance level of *p* ≤ 0.05). Pigment composition (total and individual) and antioxidant capacity (FRAP) data are presented as the least-squares mean ± standard error (SE) of N = 18 for each plant, N = 30 for each extraction method, and N = 30 for each solvent. The correlation between determined variables and their intensity was calculated with Pearson’s correlation test (95% confidence interval).

## 3. Results and Discussion

### 3.1. Determination of Carotenoids and Chlorophylls

The HPLC-DAD method, applied to hexane, acetone, and ethanol extracts obtained in this work, was able to determine 4 carotenoid (lutein, zeaxanthin, 9-*cis* lutein, and β-carotene) and 10 chlorophyll molecules (chlorophyll *b*, *b*′, *a* and *a*’, pirochlorophyll *b,* and *a*, as well as the pheophytins *b*, *b*’, *a* and *a*’) (Table 1 and Table 2). Total carotenoid content ranged from 6.02 mg 100 g^−1^ DW in *T. vulgaris* to 14.24 mg 100 g^−1^ DW in *M. communis*, with a grand mean of 9.73 mg 100 g^−1^ DW (Table 1).

Murkovic et al. [22] found 9.22 mg 100 g^−1^ of total carotenoids, composed of high levels of lutein, zexanthin and β-carotene, in the leaves of *S. officinalis*, placing it at the top of 29 different plants included in their study. The results of the current study have also shown that, regardless of the herb species, lutein was the dominant carotenoid (6.58 mg 100 g^−1^ DW grand mean). It was followed by β-carotene (2.28 mg 100 g^−1^ DW grand mean) while zeaxanthin and 9-cis lutein were present in lower amounts (0.42 and 0.45 mg 100 g^−1^ DW grand mean, respectively). The contents of individual carotenoids, with the exception of 9-*cis* lutein, and of total carotenoids were influenced by the herb species, being the highest in *M. communis* extracts and lowest in *T. vulgaris* extracts. Although, to our knowledge, no research that included the species investigated in this study has been conducted to date, these results can be compared with the research of Tattini et al. [24], where *M. communis* also had a higher content of carotenoids when compared to *P. lentiscus*. Munekata et al. [38] obtained approximately 10 mg 100 g^−1^ DW of carotenoids, extracted from *T. vulgaris*, which is similar to the results obtained in this work. On the other hand, three-fold higher total carotenoid content was measured in thyme extract in a study by Hamdan and Daood [21]; however, lutein was also found to be the dominant carotenoid. Furthermore, the concentration of total carotenoids, as determined in *T. vulgaris* (fresh weight) was 10.8 mg 100 g^−1^ and 51 mg 100 g^−1^, reported in the work of Miri et al. [39], as well as that of Karalija and Paric [40], respectively. Since carotenoid content in herbs can be affected by light and salinity [24], seasonal conditions might have caused differences in the levels of carotenoids reported in the studies published by different authors.

Chlorophyll *a*, chlorophyll *b*, and their derivatives, as detected by HPLC-DAD, are presented in Table 2. Total chlorophylls ranged from 18.60 mg 100 g^−1^ DW in *T. vulgaris* to 32.86 mg 100 g^−1^ DW in *L. nobilis*. In nature, chlorophyll molecules are present in several forms [41], with the most common being *a* and *b*. While chlorophyll *a* is characteristic of plants that are more exposed to light [42], concentrations of chlorophyll *a* and *b* depend on the plant’s age [43] and environmental factors, such as light exposure, temperature, relative humidity, etc. [44]. In the majority of *T. vulgaris* and *M. communis* extracts obtained in the current study, the content of chlorophyll *a* and its derivatives was higher, while *S. officinalis*, *L. nobilis* and *P. lentiscus* extracts, in general, contained more chlorophyll *b*. Similar findings were reported by Lafeuille et al. [34], who found 4.55 mg 100 g^−1^ of chlorophyll *b* and 0.46 mg 100 g^−1^ of chlorophyll *a* in marjoram, and 11.65 mg 100 g^−1^ of chlorophyll *b* and 13.79 mg 100 g^−1^ of chlorophyll *a* in oregano leaves.

Herb species had a significant effect on the content of all chlorophyll molecules except chlorophyll *b*. Pyrochlorophyll *a*, pheophytin *b* and *b*’, as well as total chlorophylls, were the most abundant in *L. nobilis*, chlorophyll *b′* in *S. officialis*, and pheophytin *a* and *a′* in *M. communis*. However, due to the differences in extraction rates and the degradation of individual chlorophylls that could cause an increase of pheophytin, it is difficult to relate their concentrations in extracts to their presence in herbs. In general, the average content of pheophytins was higher than the average content of chlorophylls, which could be due to the effect of the extraction temperature being applied.

**Figure 1:**
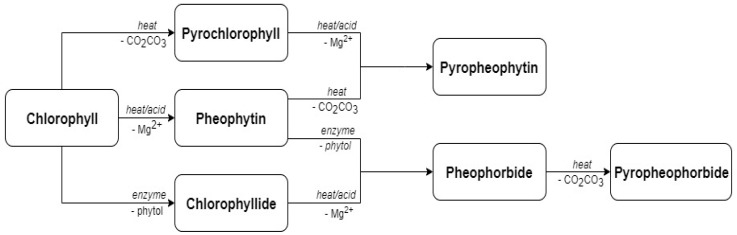
Chlorophyll degradation pathways.

Indeed, as shown in Figure 1, when exposed to temperatures above 60 °C, chlorophyll is converted to pheophytin and pyrochlorophyll, due to the replacement of the magnesium by hydrogen or a carbomethoxy group detachment, respectively [45]. In addition, prolonged heat treatment can cause the further degradation of pheophytin and pyrochlorophyll and their conversion to pyropheophytin. Conversely, enzymatic activity can cause the degradation of chlorophyll and pheophytin to pheophorbide, which can then convert to pyropheophorbide. This degradation pathway includes the detachment of the phytol group via chlorophyllase and pheophytinase, respectively, which exhibit optimal activity at 25–35 °C [46,47,48,49]. Lanfer-Marquez et al. [50] reported that among chlorophyll derivatives, both pheophytin *b* and pheophorbid *b* have been shown to be powerful antioxidants [51]. Still, due to their role in the induction of photosensitivity, Oshima et al. advise the monitoring of pheophorbides, as well as pyropheophorbide levels in food containing high levels of chlorophyll [52]. The temperature of 60 °C used in this study caused the conversion of chlorophyll to pheophytin and caused the increase in its levels. However, both pheophorbide and pyropheophorbide molecules were not detected by the applied HPLC-DAD method in any of the extracts obtained in the present study. Therefore, even though Cha et al. [53], in their PLE experiments, noted chlorophyllase inhibition and the reduction of pheophorbides at 110 °C and 135 °C, this study suggests that temperatures as low as 60 °C can be efficiently used to avoid enzymatic conversion and the formation of potentially harmful pheophorbids [52]. In addition, a temperature of 60 °C could have also had a positive effect on the content of carotenoids in the obtained extracts, as was shown in the study of Saha et al. [54], where the highest yield of lutein and β-carotene from carrot was obtained at this temperature.

Even though the HPLC determination of individual pigments presents a fairly common analytical technique for different matrices, such as olive oil and algae [55,56], data for herbs that were included in this study are very scarce. Individual pigments were determined in the work of Hamdan and Daood [21] in thyme extracts, which were found to be 7.51 and 4.13 mg/100 g^−1^ of chlorophyll *a* and *b*, and 6.53 and 1.50 mg 100 g^−1^ of pheophytin *a* and *b*, respectively. Considered individually, 80% acetone AAE of thyme, as a conventional extraction method, yielded 23.55 and 17.88 mg 100 g^−1^ of chlorophyll *a* and *b*, as well as 5.85 and 1.66 mg 100 g^−1^ of pheophytin *a* and *b* (data not shown). It is therefore obvious that, here, the obtained values for pheophytins were quite similar to those reported by Hamdan and Daood [21], while chlorophyll *a* and *b* were 3- and 4-fold higher in this study. Similar to the behavior of carotenoids, chlorophylls are influenced by environmental conditions. Ain-Lhout et al. [25] showed that the season causes significant chlorophyll decrease in *M. communis*, while Maatallah et al. [57] showed variations in chlorophyll content caused by different water deficits in two *L. nobilis* ecotypes, ranging from approximately 0.2 to 0.8 mg g^−1^.

For comparison with the HPLC-DAD method, concentrations (mg 100 g^−1^ DW) of carotenoids and chlorophylls have also been determined spectrophotometrically. The spectrophotometric method in general gave higher results when compared to the values obtained by HPLC, which was especially notable in the values of chlorophyll *a*. This result is in agreement with the work of Jacobsen and Rai [58] who found that the spectrophotometric measurement of chlorophyll *a* in aqueous samples significantly overestimated its values and gives results that are several-fold higher when compared to the HPLC results. The same relationships between the spectrophotometric and HPLC determination of chlorophyll *a* were confirmed by Pinckney et al. [59]. Nevertheless, the total carotenoid content, as determined by HPLC-DAD, strongly correlated (r = 0.724, *p* < 0.00) with the content of spectrophotometrically determined carotenoids, as well as the content of total chlorophylls as determined both by HPLC-DAD and spectrophotometrically (r = 0.776, *p* < 0.00) (data not shown). A strong correlation has also been observed between spectrophotometric data for chlorophyll *a* and the HPLC-DAD-determined total chlorophyll *a* derivatives (r = 0.762, *p* < 0.00), while spectrophotometric data for chlorophyll *b* and HPLC-DAD determined total chlorophyll *b* derivatives showed a weak correlation (r = 0.325, *p* = 0.03). This observation indicates that the spectrophotometric method can be a convenient replacement for the more demanding HPLC-DAD determination when the rapid screening of a large number of samples is required, especially if the interest is in determining the overall trends and effects. However, HPLC-DAD should be the gold standard for obtaining accurate values for individual pigment concentrations in herbal extracts. Spectrophotometric data also showed the significant influence of the plant species, with the highest levels of chlorophyll *a* and carotenoids being detected in *L. nobilis* extracts.

### 3.2. Effect of Extraction Method and Solvent

The extraction method had a significant effect on the proportions of all carotenoids, with no major differences between PLE and AAE, while UAE was generally shown to be the least effective method. The extraction method also caused significant differences in the content of all chlorophylls, except chlorophyll *a′*, *b′* and pirochlorophyll *b*, which finally reflected the content of total chlorophylls (Table 1 and Table 2). Two-fold higher levels of these compounds were extracted by PLE and AAE, in comparison with UAE. It is important to note that although higher PLE temperatures could have given higher yields [60], a temperature of 60 °C was applied to enable the better comparison of the extraction methods. Still, when comparing the applied advanced techniques, PLE was the best option, which is consistent with the study by Plaza et al. [61], where PLE was also noted as a more efficient technique than UAE.

However, when observing the effect of the interaction of the solvent and extraction method, it is noteworthy that there were no significant differences between the methods for ethanol extracts, while AAE and PLE were more efficient when hexane and 80% acetone were used (data not shown). The reason for the lowest efficiency of the UAE was most likely the polarity of the solvent since it has been shown that the extraction power of the UAE increases with the increase of solvent polarity [62]. Therefore, in this study, ethanol was shown as a solvent suitable for UAE.

The solvents with increasing polarity, as mentioned earlier, were used to cover a wide range of molecules of different polarities. The effect of the solvent was significant in the case of total carotenoids, as well as all individual carotenoids. Lutein, zeaxanthin, and 9-cis-lutein were the most effectively extracted with 80% acetone, while efficient β-carotene extraction was achieved with hexane, resulting in no significant differences in total carotenoids in the extracts obtained via these two solvents. Weakly polar and non-polar solvents dissolve β-carotene significantly better, while due to its more polar structure, solvents of higher polarity dissolve lutein [27]. Considering their polarity, the least polar carotenoids are β-carotene and lycopene, while the addition of polar groups (such as hydroxyl) to their structure (lutein and zeaxanthin) increases their polarity [63]. In addition, the solvent type significantly affected total and all individual chlorophyll levels except chlorophyll *a*’. Chlorophyll *b*, chlorophyll *b*’ and chlorophyll *a* were the most abundant in acetone and ethanol extracts, while pheophytin isomers (pheophytin *b*, pheophytin *b*’, pheophytin *a*, and pheophytin *a*’) were the most abundant in hexane (non-polar) extracts. This is due to the hydrophobic structure of pheophytin, in which, when compared to the chlorophyll molecule, the Mg^2+^ ion is absent [64]. Acetone (80%), as a more polar solvent than hexane, but being less polar than ethanol (96%), provided significantly higher yields of spectrophotometrically determined pigments. Therefore, it can be assumed that the pigments in the examined plant species are mostly of medium polarity. It is interesting to point out that, in total, carotenoids, as the second-largest group of non-polar lipid-soluble pigments, were mostly extracted with acetone as a medium polar solvent, as used in this study. Nevertheless, good solubility in acetone is due to the polar fragments of their molecules [11]. Plaza et al. [61] also reported that acetone showed higher efficiency in the extraction of carotenoids from *Chlorella vulgaris*, when compared to hexane and ethanol. Furthermore, chlorophyll *a* is better soluble in non-polar solvents and chlorophyll *b* in more polar ones [16].

**Figure 2:**
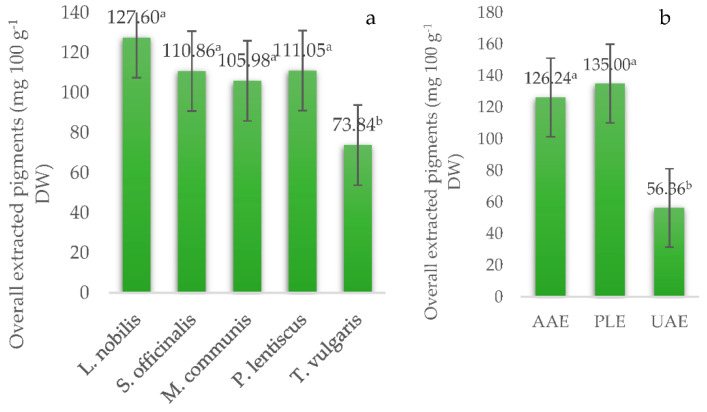
Overall extracted pigments, as determined by the HPLC-DAD method (mg 100 g^−1^ DW), in the extracts of selected Mediterranean herbs, affected by herb species (**a**) and extraction method (**b**). AAE = agitation-assisted extraction, PLE = pressurized liquid extraction, UAE = ultrasound-assisted extraction. Results are shown as mean and SE (error bar). Different letters indicate significant differences, according to Tukey’s test at *p* ≤ 0.05.

Since the purpose of this work was to increase the yield of the extraction with successive solvent extraction, the yield of the overall extracted pigments obtained by different extraction methods from particular plants was calculated and is shown in Figure 2. Successive solvent extraction is a good way to extract the components of different polarities, and the cumulative concentration value will give a comprehensive overview of their content in the examined material [63]. Yields obtained using three different solvents ranged from 73.84 mg 100 g^−1^ DW for *T. vulgaris*, which was the only species with significantly fewer overall extracted pigments, to 127.60 mg 100 g^−1^ DW for *L. nobilis*. On the other hand, even though PLE gave a slightly higher overall yield, it did not statistically differ from AAE. Both methods gave around 2.5-fold higher yields than UAE.

### 3.3. Antioxidant Capacity

In addition to being key molecules in plant photosynthesis, chlorophylls and carotenoids are also important antioxidants. [42]. Therefore, to relate the proportion of pigments to their antioxidant activity, and to compare the antioxidant potential of the examined plant extracts, a FRAP assay was performed. As can be seen from the obtained results, great variation between the extracts was present (Table 3). *M. communis* leaf extracts had a significantly higher FRAP value (31.37 mmol TE 100 g^−1^ DW), followed by the *P. lentiscus* (29.06 mmol TE 100 g^−1^ DW). Although *L. nobilis* was the richest source of pigments, its FRAP value was several-fold lower (8.29 mmol TE 100 g^−1^ DW) than that of the abovementioned species, while *S. officinalis* had a slightly higher antioxidant capacity. By determining the antioxidant capacity of 13 plants, including *S. officinalis* and *L. nobilis*, Fernandes et al. [65] also confirmed the lower FRAP value found in *L. nobilis*, when compared to *S. officinalis*. A Pearson correlation was used to determine the relationship between antioxidant capacity and the content of pigments, as determined both by HPLC-DAD and spectrophotometrically. A weak and moderate positive correlation was found between the antioxidant capacity and spectrophotometrically determined carotenoids (r = 0.294, *p* = 0.05), chlorophyll *b* (r = 0.484, *p* = 0.00), and total chlorophylls (r = 0.312, *p* = 0.04), as well as lutein (r = 0.317, *p* = 0.03) and 9-cis lutein (r = 0.418, *p* = 0.00), determined by HPLC-DAD. Furthermore, a negative correlation was obtained for antioxidant capacity and pheophytin *b* (r = −0.300, *p* = 0.05).

It is important to note that the FRAP value does not have to be correlated with the content of pigments in individual plants since its value is also affected by the amount of phenolic compounds present in plants [65,66]. Therefore, the possible reason for the obtained lower antioxidant capacity of *L. nobilis* could be its phenolic content, which is much lower than that of *M. communis*, as reported by Gião et al. [67]. These findings are in agreement with the observations of Amensour et al. [68], who concluded that *M. communis* is a good source of antioxidant components and assumed that phenolics are primarily responsible for the antioxidant activity of the extracts analyzed via DPPH, reducing power and β-carotene assays. Chryssavgi et al. [69] also proved that *P. lentiscus* and *M. communis* have great application potential since they are an excellent source of phenolics with high antioxidant capacity. Dorman et al. [70] reported that among five different species of *Lamiaceae* plants, *S. officinalis* and *Rosmarinus officinalis* had a significantly higher antioxidant capacity and, thus, the highest proportion of phenolic compounds than *T. vulgaris*; in this study, it has the lowest antioxidant capacity. These values confirm that chlorophylls and carotenoids are not the key components responsible for plant antioxidant capacity. In accordance with the results of the present study, Nobossé et al. [71] tested five antioxidant methods and documented almost the same correlation values between the FRAP method and carotenoids (r = 0.222), while the correlation with chlorophylls was higher (r = 0.588). On the other hand, they exhibited a stronger correlation of chlorophylls with antioxidant activity than phenolic compounds, and a better correlation of chlorophylls with some other antioxidant assays, e.g. ABTS (2,2′-azino-bis(3-ethylbenzothiazoline-6-sulfonic acid)) and APA (anti-peroxide activity). Although it was not statistically significant, extracts obtained with PLE showed the highest antioxidant capacity when compared to the UAE, which was again shown to be the least successful method. A study by Dahmoune et al. [72] also showed a lower antioxidant activity of *M. communis* extracts obtained by UAE and AAE when compared to microwave-assisted extraction (MAE). Moreover, Cai et al. [73] measured antioxidant activity by FRAP in purple sweet potato extracts obtained via conventional extraction (CE), UAE, and PLE, and concluded that PLE showed a higher extraction efficiency, while UAE and CE were almost equally effective. On the other hand, the antioxidant activity determined in their study by the ORAC method was higher in CE and UAE extracts. Other studies have also shown a greater efficiency of PLE over UAE, CE, and MAE in the extraction of phenolic compounds [74,75], anthocyanins [76], and flavonols [77].

The results in the present study showed that the most appropriate solvent with a significant impact on the extraction of antioxidant molecules was 80% acetone, followed by ethanol. When determining free-radical scavenging activity using DPPH, Gololo et al. [78] showed that components with antioxidative properties were more soluble in more polar solvents, which correlates well with the results obtained in this study. Numerous other studies have shown a different polar character of antioxidant molecules [79,80,81].

## 4. Conclusions

The results of this study indicated that selected plant species are rich sources of carotenoids and chlorophyll, while their distribution, concentration and antioxidant capacity depend on the species as well as applied method and extraction conditions. Therefore, *M. communis* has been shown to be the richest source of carotenoids, while the highest levels of chlorophylls were found in *L. nobilis* of chlorophyll. Furthermore, *M. communis* showed the highest antioxidant capacity and *T. vulgaris* had the lowest proportion of both examined groups of pigments, as well as antioxidant capacity. Regarding the extraction method, PLE and AAE had a similar efficiency, while UAE was shown to be the least efficient. Successive solvent extraction has proven a great diversity in the chemical structure within chlorophylls and carotenoids since each fraction obtained with solvents of different polarity (hexane, 80% acetone, and ethanol) contained a different number of individual pigments. Most compounds were of medium polarity and dissolved in 80% acetone. It was also noticed that acetone extracts were characterized by the highest antioxidant capacity according to the FRAP values. As expected, a good correlation between those pigments determined spectrophotometrically and with HPLC-DAD was confirmed by a Pearson’s matrix, although HPLC can be preferable. Screening of the composition and content of plant pigments in selected Mediterranean plants has further confirmed their potential for application in the food industry (color additives, nutraceuticals), especially for *M. communis* and *L. nobilis*, which stand out for their high pigment content and antioxidant potential. This research represents a good basis for the selection of plant species and the implementation of comprehensive research related to the optimization of the conditions of plant pigments isolation: it also serves as the primary step in the preparation of high yield extracts for further research (e.g., conversion of plant pigment extracts into more stable powder form via various encapsulation techniques).

## Figures and Tables

**Table 1:** Carotenoid content, as determined via the HPLC-DAD method (mg 100 g^−1^ DW), in the extracts of selected Mediterranean herbs, obtained through sequential solvent extraction by different extraction techniques.

Source of Variation	Lutein	Zeaxanthin	9-*cis* Lutein	β-Carotene	Total Carotenoids
**Herb species**	*p* < 0.01	*p* = 0.03	*p* = 0.13	*p* < 0.01	*p* < 0.01
*L. nobilis*	6.84 ± 1.11 ^ab^	0.43 ± 0.13 ^ab^	0.58 ± 0.18 ^a^	1.83 ± 0.41 ^bc^	9.68 ± 1.61 ^ab^
*S. officinalis*	5.85 ± 1.11 ^ab^	0.36 ± 0.13 ^ab^	0.24 ± 0.18 ^a^	2.99 ± 0.41 ^ab^	9.43 ± 1.61 ^ab^
*M. communis*	9.32 ± 1.11 ^a^	0.65 ± 0.13 ^a^	0.69 ± 0.18 ^a^	3.58 ± 0.41 ^a^	14.24 ± 1.61 ^a^
*P. lentiscus*	6.63 ± 1.11 ^ab^	0.47 ± 0.13 ^ab^	0.40 ± 0.18 ^a^	1.76 ± 0.41 ^bc^	9.26 ± 1.61 ^ab^
*T. vulgaris*	4.25 ± 1.11 ^b^	0.20 ± 0.13 ^b^	0.33 ± 0.18 ^a^	1.29 ± 0.41 ^c^	6.02 ± 1.61 ^b^
**Method**	*p* < 0.01	*p* < 0.01	*p* < 0.01	*p* < 0.01	*p* < 0.01
AAE	7.32 ± 0.86 ^a^	0.47 ± 0.10 ^a^	0.61 ± 0.14 ^a^	2.93 ± 0.31 ^a^	11.33 ± 1.25 ^a^
PLE	8.97 ± 0.86 ^a^	0.59 ± 0.10 ^a^	0.58 ± 0.14 ^a^	3.13 ± 0.31 ^a^	13.27 ± 1.25 ^a^
UAE	3.45 ± 0.86 ^b^	0.21 ± 0.10 ^b^	0.15 ± 0.14 ^b^	0.78 ± 0.31 ^b^	4.58 ± 1.25 ^b^
**Solvent**	*p* < 0.01	*p* = 0.04	*p* < 0.01	*p* < 0.01	*p* < 0.01
Hexane	6.81 ± 0.86 ^a^	0.42 ± 0.10 ^ab^	0.27 ± 0.14 ^b^	5.94 ± 0.31 ^a^	13.45 ± 1.25 ^a^
Acetone-80%	8.81 ± 0.86 ^a^	0.56 ± 0.10 ^a^	1.01 ± 0.14 ^a^	0.59 ± 0.31 ^b^	10.97 ± 1.25 ^a^
Ethanol-96%	4.11 ± 0.86 ^b^	0.29 ± 0.10 ^b^	0.06 ± 0.14 ^b^	0.31 ± 0.31 ^b^	4.76 ± 1.25 ^b^
**Grand mean**	6.58	0.42	0.45	2.28	9.73

AAE = agitation-assisted extraction, PLE = pressurized liquid extraction, UAE = ultrasound-assisted extraction. Total carotenoid content refers to the sum of lutein, zeaxanthin, 9-*cis* lutein, and β-carotene. Results are expressed as mean ± SE. Different letters within columns indicate significant differences according to Tukey’s test at *p* ≤ 0.05.

**Table 2:** Chlorophyll content, as determined by the HPLC-DAD method (mg 100 g^−1^ DW), in the extracts of selected Mediterranean herbs obtained through sequential solvent extraction by different extraction techniques.

Source of Variation	Chl *b*	Chl *b’*	Piro *b*	Chl *a*	Chl *a’*	Piro *a*	Phe *b*	Phe *b’*	Phe *a*	Phe *a’*	Total Chlorophyll
**Herb species**	*p* = 0.12	*p* < 0.01	*p* < 0.01	*p* < 0.01	*p* < 0.01	*p* < 0.01	*p* < 0.01	*p* < 0.01	*p* < 0.01	*p* = 0.02	*p* = 0.04
*L. nobilis*	4.72 ± 1.52 ^a^	0.36 ± 0.22 ^ab^	0.11 ± 0.19 ^bc^	3.11 ± 1.34 ^ab^	0.04 ± 0.11 ^b^	0.22 ± 0.04 ^a^	10.47 ± 1.36 ^a^	8.63 ± 1.18 ^a^	4.22 ± 1.25 ^c^	0.97 ± 0.31 ^ab^	32.86 ± 4.45 ^a^
*S. officinalis*	4.99 ± 1.52 ^a^	0.95 ± 0.22 ^a^	0.50 ± 0.19 ^ab^	4.77 ± 1.34 ^a^	0.44 ± 0.11 ^a^	0.00 ± 0.04 ^b^	4.70 ± 1.36 ^bc^	4.45 ± 1.18 ^bc^	5.77 ± 1.25 ^bc^	0.96 ± 0.31 ^ab^	27.52 ± 4.45 ^ab^
*M. communis*	3.22 ± 1.52 ^a^	0.00 ± 0.22 ^b^	0.00 ± 0.19 ^c^	0.22 ± 1.34 ^b^	0.00 ± 0.11 ^b^	0.00 ± 0.04 ^b^	1.15 ± 1.36 ^c^	2.66 ± 1.18 ^c^	12.04 ± 1.25 ^a^	1.80 ± 0.31 ^a^	21.09 ± 4.45 ^ab^
*P. lentiscus*	2.31 ± 1.52 ^a^	0.34 ± 0.22 ^ab^	0.00 ± 0.19 ^c^	0.51 ± 1.34 ^b^	0.04 ± 0.11 ^b^	0.09 ± 0.04 ^ab^	6.97 ± 1.36 ^ab^	7.48 ± 1.18 ^ab^	8.87 ± 1.25 ^ab^	1.15 ± 0.31 ^ab^	27.75 ± 4.45 ^ab^
*T. vulgaris*	4.51 ± 1.52 ^a^	0.44 ± 0.22 ^ab^	0.66 ± 0.19 ^a^	4.78 ± 1.34 ^a^	0.11 ± 0.11 ^b^	0.00 ± 0.04 ^b^	1.48 ± 1.36 ^c^	2.36 ± 1.18 ^c^	3.68 ± 1.25 ^c^	0.58 ± 0.31 ^b^	18.60 ± 4.45 ^b^
**Method**	*p* = 0.01	*p* = 0.44	*p* = 0.20	*p* = 0.03	*p* = 0.06	*p* = 0.01	*p* < 0.01	*p* < 0.01	*p* < 0.01	*p* < 0.01	*p* = 0.00
AAE	5.33 ± 1.18 ^a^	0.52 ± 0.17 ^a^	0.45 ± 0.15 ^a^	4.21 ± 1.04 ^a^	0.25 ± 0.09 ^a^	0.11 ± 0.03 ^a^	5.40 ± 1.05 ^ab^	4.69 ± 0.91 ^b^	8.61 ± 0.97 ^a^	1.17 ± 0.24 ^ab^	30.75 ± 3.44 ^a^
PLE	4.01 ± 1.18 ^ab^	0.44 ± 0.17 ^a^	0.26 ± 0.15 ^ab^	1.57 ± 1.04 ^b^	0.04 ± 0.09 ^a^	0.07 ± 0.03 ^ab^	6.53 ± 1.05 ^a^	7.84 ± 0.91 ^a^	9.46 ± 0.97 ^a^	1.52 ± 0.2^4 a^	31.73 ± 3.44 ^a^
UAE	2.51 ± 1.18 ^b^	0.29 ± 0.17 ^a^	0.06 ± 0.15 ^b^	2.26 ± 1.04 ^ab^	0.10 ± 0.09 ^a^	0.00 ± 0.03 ^b^	2.93 ± 1.05 ^b^	2.82 ± 0.91 ^b^	2.67 ± 0.97 ^b^	0.58 ± 0.24 ^b^	14.21 ± 3.44 ^b^
**Solvent**	*p* < 0.01	*p* < 0.01	*p* < 0.01	*p* < 0.01	*p* = 0.12	*p* < 0.01	*p* < 0.01	*p* < 0.01	*p* < 0.01	*p* < 0.01	*p* < 0.01
Hexane	0.05 ± 1.18 ^c^	0.00 ± 0.17 ^b^	0.00 ± 0.15 ^c^	0.15 ± 1.04 ^b^	0.05 ± 0.09 ^a^	0.15 ± 0.03 ^a^	9.31 ± 1.05 ^a^	8.20 ± 0.91 ^a^	8.80 ± 0.97 ^a^	1.63 ± 0.24 ^a^	28.34 ± 3.44 ^a^
Acetone-80%	7.45 ± 1.18 ^a^	0.60 ± 0.17 ^ab^	0.52 ± 0.15 ^a^	4.24 ± 1.04 ^a^	0.10 ± 0.09 ^a^	0.04 ± 0.03 ^b^	3.07 ± 1.05 ^b^	4.48 ± 0.91 ^b^	8.39 ± 0.97 ^a^	1.15 ± 0.24 ^a^	30.03 ± 3.44 ^a^
Ethanol-96%	4.35 ± 1.18 ^b^	0.65 ± 0.17 ^a^	0.25 ± 0.15 ^ab^	3.64 ± 1.04 ^a^	0.23 ± 0.09 ^a^	0.00 ± 0.03 ^b^	2.49 ± 1.05 ^b^	2.66 ± 0.91 ^b^	3.56 ± 0.97 ^b^	0.49 ± 0.24 ^b^	18.31 ± 3.44 ^b^
**Grand mean**	3.95	0.42	0.26	2.68	0.13	0.06	4.95	5.12	6.92	1.09	25.56

AAE = agitation-assisted extraction, PLE = pressurized liquid extraction, UAE = ultrasound-assisted extraction. Chl = chlorophyll, Piro = pirochlorophyll, Phe = pheophytin. Total chlorophyll content refers to the sum of Chl (*b*, *b′*, *a*, *a′*), Piro (*b*, *a*), Phe (*b*, *b′*, *a*, *a′*). Results are expressed as mean ± SE. Different letters within columns indicate significant differences, according to Tukey’s test at *p* ≤ 0.05.

**Table 3:** Spectrophotometric determination of pigments (mg 100 g^−1^ DW) and antioxidant capacity (mmol TE 100 g^−1^ DW) in extracts of selected Mediterranean herbs, obtained through sequential solvent extraction by different extraction techniques.

Source of Variation	Car	Chl *a*	Chl *b*	Total Chl	FRAP
**Herb species**	*p* = 0.01	*p* = 0.04	*p* = 0.20	*p* = 0.07	*p* < 0.01
*L. nobilis*	8.56 ± 0.65 ^a^	24.28 ± 2.34 ^a^	10.07 ± 1.23 ^a^	34.35 ± 3.34 ^a^	8.29 ± 3.98 ^c^
*S. officinalis*	7.40 ± 0.65 ^ab^	16.76 ± 2.34 ^ab^	9.29 ± 1.23 ^a^	26.05 ± 3.34 ^a^	12.28 ± 3.98 ^b^
*M. communis*	7.40 ± 0.65 ^ab^	14.38 ± 2.34 ^ab^	6.31 ± 1.23 ^a^	20.70 ± 3.34 ^a^	31.37 ± 3.98 ^a^
*P. lentiscus*	5.64 ± 0.65 ^b^	16.27 ± 2.34 ^ab^	6.72 ± 1.23 ^a^	22.98 ± 3.34 ^a^	29.06 ± 3.98 ^ab^
*T. vulgaris*	5.32 ± 0.65 ^b^	13.89 ± 2.34 ^b^	8.10 ± 1.23 ^a^	21.99 ± 3.34 ^a^	4.38 ± 3.98 ^c^
**Method**	*p* < 0.01	*p* = 0.01	*p* = 0.15	*p* = 0.02	*p* = 0.23
AAE	7.39 ± 0.50 ^a^	19.22 ± 1.81 ^a^	7.91 ± 0.95 ^a^	27.13 ± 2.59 ^ab^	17.06 ± 3.08 ^a^
PLE	8.65 ± 0.50 ^a^	20.26 ± 1.81 ^a^	9.57 ± 0.95 ^a^	29.83 ± 2.59 ^a^	21.02 ± 3.08 ^a^
UAE	4.55 ± 0.50 ^b^	11.87 ± 1.81 ^b^	6.81 ± 0.95 ^a^	18.68 ± 2.59 ^b^	13.16 ± 3.08 ^a^
**Solvent**	*p* < 0.01	*p* < 0.01	*p* < 0.01	*p* < 0.01	*p* < 0.01
Hexane	6.49 ± 0.50 ^b^	14.97 ± 1.81 ^b^	1.47 ± 0.95 ^c^	16.44 ± 2.59 ^b^	0.77 ± 3.08 ^c^
Acetone-80%	10.20 ± 0.50 ^a^	24.27 ± 1.81 ^a^	14.88 ± 0.95 ^a^	39.14 ± 2.59 ^a^	37.00 ± 3.08 ^a^
Ethanol-96%	3.89 ± 0.50 ^c^	12.11 ± 1.81 ^b^	7.95 ± 0.95 ^b^	20.07 ± 2.59 ^b^	13.46 ± 3.08 ^b^
**Grand mean**	6.86	17.12	8.10	25.21	17.08

AAE = agitation-assisted extraction, PLE = pressurized liquid extraction, UAE = ultrasound-assisted extraction, Car = carotenoid, Chl = chlorophyll. Results are expressed as mean ± SE. Total chlorophyll content refers to the sum of Chl *a* and Chl *b*. Different letters within columns indicate significant differences according to Tukey’s test at *p* ≤ 0.05.

## Data Availability

All the data available is in the manuscript.
